# Exploring the initiation of fiber fuse

**DOI:** 10.1038/s41598-019-47911-0

**Published:** 2019-08-12

**Authors:** Qirong Xiao, Jiading Tian, Ping Yan, Dan Li, Mali Gong

**Affiliations:** 1 Key Laboratory of Photonic Control Technology (Tsinghua University), Ministry of Education, Haidian District, Being, 10084 China; 2 State Key Laboratory of Precision Measurement Technology and Instruments, Department of Precision Instrument, Tsinghua University, Haidian District, Beijing, 100084 China

**Keywords:** Fibre optics and optical communications, Phase transitions and critical phenomena

## Abstract

We report an investigation of conditions for the initiation of fiber fuse (IFF), a kind of catastrophic damage that troubles all kinds of optical fibers, in silica-based optical fibers. The fibers of different chemical compositions were processed and tested in controlled conditions without mechanical damages before the IFF. For all the fibers of IFF, the same correlation between the critical temperatures and the optical powers transmitted therein was revealed for the first time. The fibers of different chemical compositions exhibited different resistances to the IFF under the threshold powers for propagation of fiber fuses. The results offered promise for predicting fiber fuses in optical fiber systems, which could facilitate avoiding catastrophic losses. They could direct the optimization of fiber production technologies for suppressing the damages, as well as open a new path towards controlled utilization of fiber fuse in in-fiber microstructure fabrication.

## Introduction

Fiber fuse, having been observed in all kinds of optical fibers known to date^1–18^, is a famous kind of laser-induced damages. Its name came from that the propagation of fiber fuse (PFF) resembles a blasting fuse^1,11^ moving along fibers, usually leaving a series of bullet-shaped cavities (several-micrometer diameter) inside. Fiber fuse can happen spontaneously in fiber systems without a warning; it is uncertain to the users of the systems when it will happen. It proves threatening both continuous-wave and pulse^11,12^ laser systems. But it contrasts with the conventional laser-induced damages^19^ for its much lower threshold optical power (~MW/cm2 for single-mode glass fibers^2,6–8,12^ or ~kW/cm^2^ for polymer fibers^5,9^). The very-low thresholds allow fiber fuse, once it is initiated, to destroy the whole fiber systems. For example, fiber fuse has become the most common and catastrophic kind of damages in high-power fiber laser systems^6^, causing uncharted economical losses. As the use of optical fibers prevails in many branches of physical researches and engineering, preventing spontaneous fiber fuses becomes an urgent request.

In literature, fiber fuse for research was only produced by unmeasurable means, such as deliberately damaging the laser-on fibers via collision to metals^4,11^, immersion in metal powders^5,18^ or shocking in electric arcs^10,17^. However, these complicated circumstances could include multiple mechanisms^20,21^ that induced damages to fibers that triggered the fiber fuses. This presented huge difficulties in quantitatively verifying the reasons for fiber fuse, which was the real threat in optical fiber systems. Despite the limited experimental capabilities to measurement, an assumption was that fiber fuse has an evolving timeline that accidental high temperatures (i.e. 1050 °C)^8,10,11^ in optical fibers lead to uncontrolled increase of optical absorption, which further increase the temperature and finally decompose the fiber cores in a chain-reaction manner. Empirically, the assumption agreed with that fiber fuses were more often observed in fiber systems transmitting high average optical powers, where thermal effects accompany light-matter-interactive devices therein^6,12^. However, no specific temperature conditions were experimentally verified to trigger fiber fuse yet. The assumed timeline of fiber fuse was so far with only limited and indirect links to experimental evidence.

In fact, like in many complex physical phenomena, we can suppose a period in the timeline of fiber fuse as the initiation of fiber fuse (IFF). Logically, this IFF happens before the materials of fiber inevitably evolve into the following catastrophic damage. That is, for example, the IFF is the period before the assumed surge of optical absorption at the assumed 1050 °C; the assumed surge of optical absorption, which was not the concern of this paper, will be regarded as part of the following fiber fuse itself. Although it is difficult to observe directly the microscopic process of the IFF, the IFF can be a transient process according to the experimentally experienced absence of macroscopic warning signals for fiber fuse. For this transient process, it is expected that there are critical conditions. The critical conditions can provide useful information about the deterministic physical mechanisms of the IFF. Practically, it is possible to reveal the critical conditions, should they link to slowly varying environmental parameters that can be measured at the beginning of the macroscopically observed fiber fuse. That is, for example, verifying deterministic temperature conditions to trigger fiber fuse is important for understanding the IFF, should the IFF includes a temperature-dependent microscopic process. For these experimental verifications, imitating a spontaneous fiber fuse in experiment is very important. Non-spontaneous fiber fuses, such as by mechanically damaging the fibers or other drastic changes of environmental parameters, may include more complicated mechanisms and cause difficulties in verifying the critical conditions. In contrast, a spontaneous fiber fuse happens after the microscopic environment evolve into the IFF with smoothly varying experimental parameters. If deterministic values can be found for the parameters, the critical conditions for the IFF may be thus verified.

In this paper, the IFF were for the first time triggered in controlled and measurable environments and conditions. The required critical temperatures for the IFF were found to increase while the transmitted optical powers in the fibers decreased. A quantitative correlation between the critical temperature and optical power conditions for the IFF was clearly revealed. Meanwhile, the conditions for IFF varied significantly in fibers of different chemical compositions (i.e. minimum critical temperatures from 836 to 973 °C). The results suggested that the conditions for IFF were determined by activation energies that could relate to temperature-dependent equilibrium of oxygen diffusion in amorphous silica. The correlation could offer a feasible way to predict the IFF in typical fiber systems by testing the fibers before usage. It could also present new requirements for fiber design and production technologies to suppress the IFF. Furthermore, the experiment revealed that delicately adjusting the critical conditions for the IFF could result in different characteristics of the leading in-fiber cavities formed after the IFF. The forming of the leading in-fiber cavities, observed via monitoring the beam profile of the fibers, could last more than a second without PFF. These findings inspire a controllable and efficient method to fabricate and integrate microstructures of designable characteristics in fibers.

## Results

### Experimental design and the outcomes

**Figure 1:**
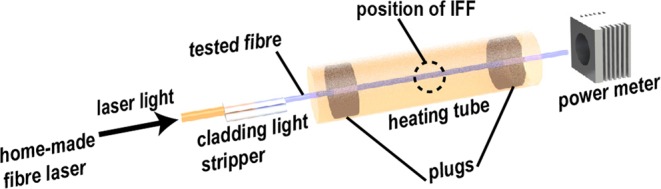
Experimental setup for triggering the IFF.

Previous studies suspected that fiber fuse in silica fibers related to a temperature-induced absorption^8,10,11^. It was assumed that the absorption would surge at around 1050 °C and sustain the PFF^22,23^. However, such temperature was only experimentally measured with surging losses of output powers of heated fibers, but with no fiber fuse^11,24^. To date, the assumed critical conditions have neither been experimentally verified nor theoretically rigorously correlated to the IFF. For verifying the critical conditions for IFF, the environment of triggering the initiation must be controlled and measurable. Meanwhile, high enough conditions must be provided to trigger the initiation of fiber fuse to avoid false negative results. Neither deliberately contacting the fiber ends with metals nor electric-arc shocking could satisfy these requirements. In this paper, the experimental setup shown in Fig. 1 was built to trigger the IFF under controlled conditions. 4 kinds of active fibers (numbered fiber 1–4) and 2 kinds of passive fibers (numbered fiber 5 and 6) were tested (the specifications for the 6 fibers shown in Supplementary Note 1 and Supplementary Table S5, the test of chemical compositions in Supplementary Note 2 and Supplementary Table S6). For controlling the parameters, these fibers were all single-mode silica fibers of core diameters less than 10 μm and (inner) cladding diameters around 130 μm. These parameters were also the most commonly used in previous experimental studies on fiber fuse^1,2,5,7–11^. Each time a home-made 1064-nm continuous-wave fiber laser injected an amount of optical power into the fiber. The maximum output power of the fiber laser was 70 W before cladding light stripper; the maximum optical powers that entered the tested fibers could be 50~60 W, which were the highest continuous-wave power ever applied in the study of fiber fuse in those fibers. The highest power density therein could approximate 58.9 MW/cm^2^ in the active fibers and 114.4 MW/cm^2^ in the passive fibers (calculation of the power densities is shown in Supplementary Note 1). It was around 40 times larger than that of the previously failed attempt^24^, which we believed was the closest one to reveal the critical conditions for the IFF.

**Figure 2:**
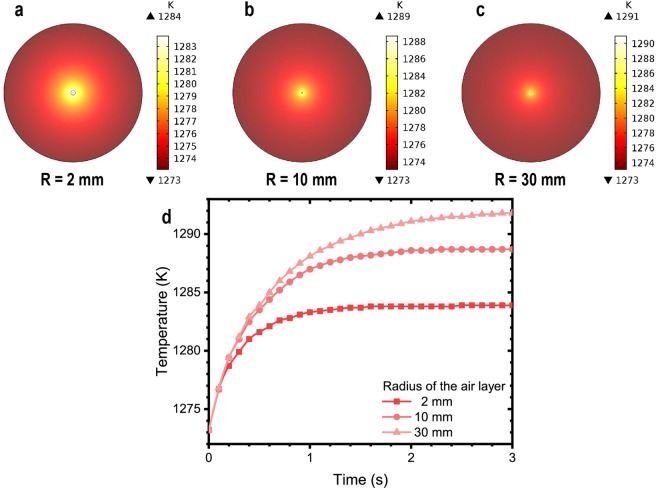
Simulated temperature field around the fiber. (**a**–**c**) Are of different radiuses of the air layers R = 2, 10 and 30 mm, respectively. (**d**) The highest temperatures of the fiber increasing with time.

As uniform temperature environment was needed, the fiber was heated in a nearly-closed tube furnace, where the temperature could reach 1200 °C. This temperature was also the highest ever applied in the study of fiber fuse and covered the assumed 1050 °C surge point in literature. At this point, a concerning problem was the possible optical absorption that could change the temperature of the fibers during the heating. If optical absorption would surge after the temperature reached around the 1050 °C as assumed, it might also cause significant temperature differences between the fiber and the environment before the IFF. That would make measuring the critical temperature for the IFF impossible. We used numerical simulations to validate that our experimental setup could provide uniform and measurable conditions for the IFF. A heat transfer model was built using the experimental parameters of the fibers and considering maximum possible heat effect by the experimentally observed optical power losses before the IFF (as shown later). As can be seen in Fig. 2, the dynamic temperatures of the core of the fibers before the IFF (or any material phase changes that inevitably result in it) would reach stable values in short seconds, which was sufficiently fast for following the heating process. Meanwhile, the stable temperature differences between the core of the tested fibers and the environmental temperatures in the furnace were less than 10 °C considering an isotropic air boundary of 2-mm radius in cross section of the heating tube, or less than 20 °C considering that of even 30-mm radius (which was the radius of the tube furnace). Moreover, the temperature gradient on the fiber itself, which was the factor that could directly influence the IFF, was less than 1 °C. Note that these results were based on that the experimentally observed optical power losses were all absorbed and converted into heat. However, as shown later, the optical power losses could be largely attributed to leakage instead of absorption. These results suggested that the actual temperature could only be negligible in the experiment. That is, the measured temperatures in the tube furnace could approximate those of the fibers. Additionally, as the distributed absorption was negligible and could not impose significant temperature changes on fibers, the distributed absorption itself could not assume responsibility for the IFF in a way that causes the temperature to uncontrollably increase. The real cause should be verified otherwise by the experimental measurement of the critical conditions for the IFF.

By the above setup, we expected fiber fuses to be triggered during the heating. As we suppose that the IFF be transient process right before the macroscopically observed fiber fuse, the happening of the IFF could be determined by the appearance of fiber fuse immediately. Measuring the critical temperatures in tube for the happening of fiber fuse was of our experimental interest, as those critical temperatures should be the critical temperatures for the IFF. At first, we expected that the IFF happen before the maximum temperature was reached; however, the IFF did not always happen. The tested passive fibers all survived at 1200 °C. The output powers of those fibers were invariant during the heating process. After we terminated the test and pulled out of the fibers, the fibers seemed to be ‘intact’, as if they never experienced the test. Contrarily, the IFF happened in the tested active fibers at much lower temperatures, from 836 to 1085 °C. These outcomes are unexpected, especially considering the previous assumption of the 1050 °C surge point in passive fibers of the tested kinds. When the IFF happened, the output powers of the fibers would plummet immediately. After the IFF, it was found that the positions of the IFF (the first in-fiber cavities) were always around the midpoints of the heated fibers. These positions, considering the symmetrical furnace structure, were under the highest temperatures in the furnace, while the temperature gradients therein should be trivial. This phenomenon suggests that high temperature has deterministic relation with the IFF.

### The non-IFF results and the output power losses before the IFF

The tested passive fibers survived in maximum testing conditions (58.4 or 114.4 MW/cm^2^ and 1200 °C, the power densities calculated in Supplementary Note 1) without IFF. The fibers even worked stably in 5-minute durations at the maximum temperatures without any optical power losses. Moreover, it was noticed that IFF could be triggered in the tested passive fibers if their polymer coating layers were not completely removed and were heated in the furnace. Considering that the residual coating layers may increase local temperatures on the tested passive fibers during the heating, as well as the IFF results (shown later), it is reasonable to presume that the IFF in the passive fibers require higher critical temperatures than 1200 °C.

The tested active fibers, in the cases of non-IFF, all shared a similar pattern: the output powers slowly diminished to zero while they were heated. The losses of output powers could become noticeable at high temperatures, i.e. 900 °C. Meanwhile, the output powers could fluctuate stochastically. In the first look, the diminutions could have been carelessly ascribed to a temperature-dependent absorption. If this were true, measuring the critical temperatures for IFF would be impossible, as the influence of the absorption were hard to be evaluated for every sample. However, we found that if the in-furnace temperatures were maintained at high values, i.e. 1000 °C, the output powers would keep diminishing. Moreover, after the fibers were pulled out in the middles of the heating, the output powers would not recover to their initial values. These phenomena suggested that the optical power losses should not be attributed to temperature-dependent absorption. In fact, the unrecoverable output power was noticed by previous research^24^ but seemed to remain underappreciated in the following studies.

**Figure 3:**
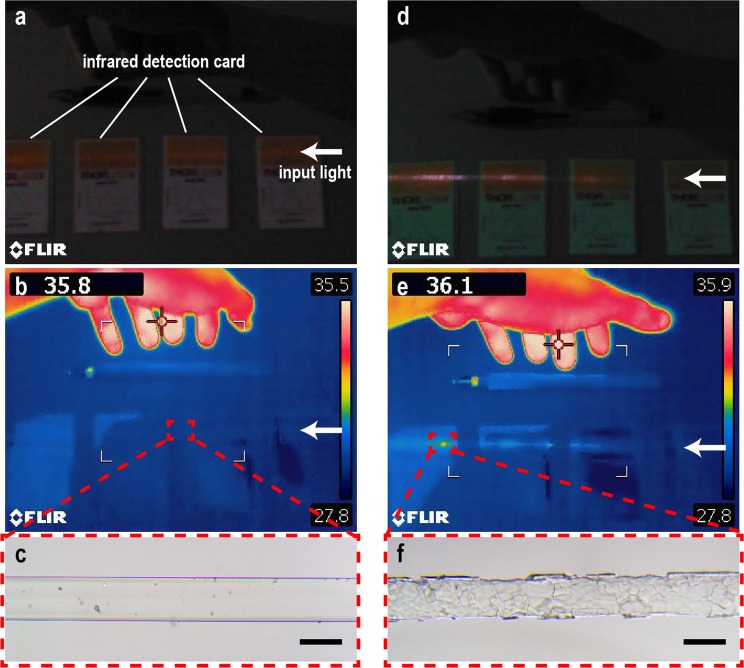
Fibers of which the output powers diminished in the heating process but without the IFF. (**a**–**c**) Are of the fiber of a loss of 1.94 W; (**d**–**f**) are of a loss of 5.78 W. (**a** and **d**) Photos of the respective fibers being input with their original input powers (8 W). The infrared detection cards uncover leaked laser light in both fibers. (**b** and **e**) Thermal images of the fibers. Human hands were included in the images to contrast with the temperature increases of the fibers. The numbers shown at the top-left of the images are the highest temperatures in the images in °C. (**c** and **f**) Micrographs of the fibers; bars are of 130-μm length.

In some cases when the optical power losses were large, it could be observed with infrared detection cards that laser light was leaked out along the heated sections of the fibers. Using an infrared thermal camera, discrete sites of temperature increases could be sometimes found on the fibers, which suggested imperfect structures on the fibers. Micrographs of the fibers would further confirm that. For example, Fig. 3 showed results from two samples (of fiber 3) that were both injected with 8-W optical powers and heated but pulled out of the furnace at different time. In one sample shown in Fig. 3a–c, the persisting optical power diminution was 1.94 W; in another shown in Fig. 3d–f, it was 5.78 W. The infrared detection card saw leakage of laser light in both fibers; the leakage in the latter one was more than that in the former one. Thermal images showed that there were hot spots on the latter one (Fig. 3e); micrographs showed that the surface of the fiber cracked (Fig. 3f) at the hot spots. Contrarily, the former one of small output power loss had no increase of temperature under thermal images, which suggested that the loss of output power was not due to a uniform temperature-induced absorption in the materials of the tested fibers.

The above phenomena indicate that the diminutions of output powers should not be originally attributed to temperature-induced absorption of the materials of the fibers. Considering all the results, the diminutions should be mainly caused by the collapse of refractive-index (RI) profiles of the fibers in the heating processes. This collapse could result from dopant diffusions in the high temperatures. It was not only influenced by the values of the temperature, but by the time that the temperatures were maintained. The confinement of the light in core was relaxed, thus causing the observed leakage. As this change is a dynamic process, it also explains the fluctuations of output power in high temperatures.

### The critical conditions for the IFF

**Figure 4:**
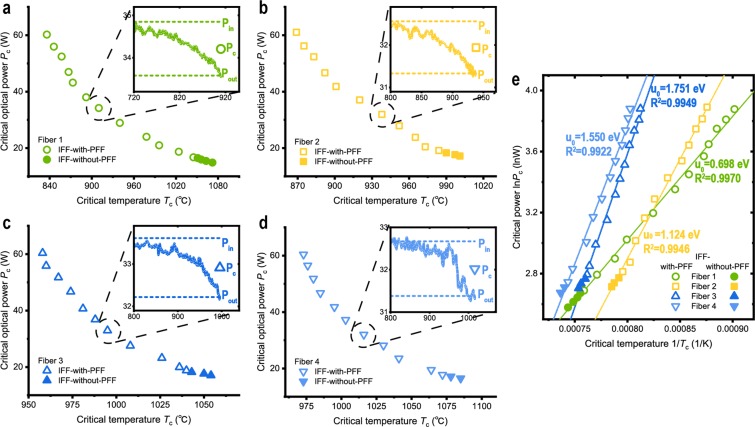
The critical optical powers *P*_*c*_ varying with the critical temperatures *T*_*c*_ for the IFF. Subplot (**a**–**d**) are *P*_*c*_ versus *T*_*c*_ (in °C) results of fibre 1–4, respectively. Insets are examples of the real-time output powers recorded while the temperatur**e**s were increasing. (**e**) linear correlation between $\mathrm{ln}\,{P}_{c}$ and 1/*T*_*c*_ (in 1/K), wherein the straight lines are the least-square linear fits and *u*_0_ calculated by Eq. 1.

In the tested active fibers, the IFF could be triggered in the experiment when the input optical powers were larger than around 18 W. Before the IFF, the output powers started to fluctuate and diminish noticeably in high temperatures, i.e. 150 °C lower than the critical conditions for IFF. Examples of these fluctuations and diminutions of output power are shown in the insets of Fig. 4a–d. The IFF would be triggered when the total diminutions reached around 1 W. Considering the above non-IFF results, the diminutions of output powers here could not impose any considerable increases on the temperatures of the fibers. Therefore, the last-recorded temperatures inside the furnace before the IFF are used as critical temperatures *T*_*c*_ for the IFF. The critical optical powers *P*_*c*_ for the IFF are determined by $\sqrt{{P}_{in}{P}_{out}}$ (Methods). Therein, *P*_*in*_ is the input power (output power recorded at the beginning of the heating process) and *P*_*out*_ the output power (recorded right before the IFF) of the fibers. Accordingly, Fig. 4a–d illustrate the variations of the critical conditions for the IFF in these fibers.

As can be seen, a similar pattern emerges as higher *P*_*c*_ corresponds to lower *T*_*c*_. The critical temperatures span a range of around 100 °C to 250 °C for each kind of the fibers. Moreover, a transformation of coordinates reveals a prominent liner correlation between $\mathrm{ln}\,{P}_{c}$ and 1/*T*_*c*_ (wherein *T*_*c*_ in Kelvin), as is shown in Fig. 4e. We propose that this correlation can be expressed as

1 $$\mathrm{ln}\,{P}_{c}=\frac{{u}_{0}}{{k}_{B}}\times \frac{1}{{T}_{c}}+\,\mathrm{ln}\,\gamma ,$$

where ln is sign of logarithm, *γ* an invariant parameter with the same dimension as *P*_*c*_, *k*_*B*_ the Boltzmann constant and *u*_0_ a parameter with dimension of energy. In this way, Eq. 1 can be also transformed into

2 $$\gamma ={P}_{c}\exp (-\frac{{u}_{0}}{{k}_{B}{T}_{c}}).$$

The exponent in Eq. 2 reminisces a temperature-dependent equilibrium of chemical reactions conforming to Arrhenius law. In this sense, *u*_0_ plays the role of the activation energy of the reactions, and the exponent in Eq. 2 should be the concentration of the products of the reactions. In an average sense, each unit of the products could absorb an amount of energy of light. Thus, the absorbed energy was proportional to the concentration of the products, which gives physical meaning to Eq. 2. In this way, the invariant product of *P*_*c*_ and the exponent in Eq. 2 indicates that the same threshold destructive energies are required for the IFF in the same fibers. In hindsight, this relation explains the known fact that the speed of PFF depends linearly on the optical power^1–12^. That fact should suggest each unit of length of the fiber-fused fiber has endured the same amount of optical power during fiber fuse. As can be seen, Eq. 1 was not representing the macroscopically observed output power losses. The critical conditions for the IFF here show no connection to the assumed distributed absorption above in our discussion around Figs 2 and 3. That is, the IFF was not preceded by an uncontrollably increasing optical absorption. Not that at this point the results here are, in fact, compatible with the assumption of previous studies that an increasing absorption lead to fiber fuse after temperature reaches certain points. The results here neither prove nor negate possible absorptions that could take place after the critical conditions were met. The critical conditions for the IFF here logically precede any possible increasing absorptions that could increase the local temperatures inside the fibers.

Our examination showed that strong signs of dangling bonds arose in the post-fiber-fuse fibers (Supplementary Fig. S1). Previous studies assumed that germanium-related defects^8,22–25^ participate in the evolving process of fiber fuse (the logic of that assumption is viewed in Supplementary Note 3). If the forming of the dangling bonds could already be significant before the final damage of fiber fuse, *u*_0_ could link to the formation of these bonds. Nevertheless, it is hard to match up *u*_0_ with the formation energies of single kinds of germanium-related defects^26–32^. Most dopant-related defects in the tested fibers (Supplementary Table S6), including germanium and aluminum ones, have larger known formation energies^26–30^ than *u*_0_ experimentally revealed here. Moreover, it is hard to find a direct link between *u*_0_ and the concentration of single kind of dopants in the tested fibers. In contrast, it is noticed that the diffusion of oxygen, as a well-known chemical process in non-crystalline doped silica resulting in oxygen deficient centers (OHCs), can have lower energy barriers being from 0.6 to 1.5 eV^28–32^. The energy barriers are subtly influenced by metal-silicate and silicon-silicate surfaces and thus can vary significantly in the substances of different chemical structures. They are also more interactive with the photons of the laser light (1.165 eV) and can produce oxygen that filled the in-fiber cavities. Considering this, it is reasonable to speculate that the IFF originate from drastic oxygen diffusion in the doped silica substances under temperature-dependent equilibrium. However, owing to the limited experimental capacity, it was hard to monitor the diffusion of oxygen during the IFF. Therefore, this speculation can be further verified in future works with suitable experimental approaches.

### The evolution of fiber fuse right after the IFF

**Figure 5:**
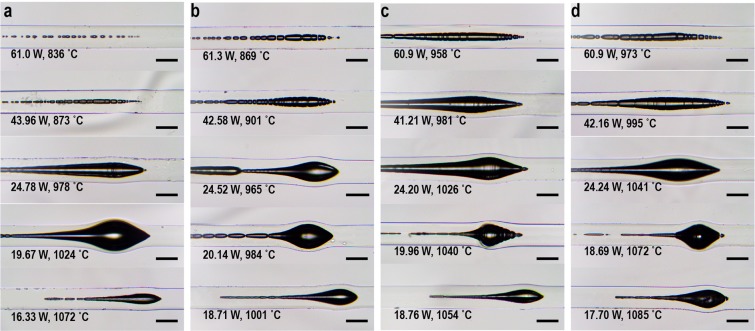
Micrographs of the positions of IFF in the tested fibers. (**a**–**d**) Are of tested fiber 1–4, respectively. Laser light was input from left to right when the IFF were triggered. In each subplot shows the input power and the temperature at which the IFF happened. The bars are of 130 μm length.

In the previous studies, the in-fiber cavities formed at the beginnings of fiber fuses were hard to be observed owing to the destructive methodologies to trigger the IFF (exception as Todoroki^10^ where delicate parameters of electric arcs were applied). In this experiment, the stable environment that inflicted no mechanical damages on the tested fibers preserved the leading in-fiber cavities at the positions of the IFF. This offers great opportunities for observing the morphologies of these cavities. As the leading in-fiber cavities formed at the beginning of the fiber fuses right after the IFF, they may provide information closely related to the IFF and more thorough understandings of the IFF. For doing this, the procedures elaborated in Methods were taken. Consequently, Fig. 5a–d show optical micrographs of the positions of the IFF in fibers 1–4, respectively. In each kind of fibers, the leading cavities are larger and thicker in the IFF-with-PFF when the input powers were lower. But when the input powers were low enough that fiber fuse could not propagate after initiation, the leading cavities shrank and became stringent. Besides, the morphologies of the leading cavities slightly differ among the kinds of fibers. The trend can be seen more clearly at high input powers around 60 W. At the beginning positions of the IFF, the cavities tend to scatter in the fibers of lower *u*_0_, while they tend to huddle together to form a long leading cavity in the fibers of higher *u*_0_.

Previous studies proposed that a fluid instability could dominate the separation of gas-phase in liquid silica formed during the PFF and determines the intervals among the generated in-fiber cavities^22^. The morphologies of the leading in-fiber cavities in Fig. 5 here evidence the instability^33–36^. However, the influence imposed by the fluid instability could be more complex, as the in-fiber cavities are not periodically separated nor longitudinal symmetrical. In fact, as the laser were input only from one side, the in-fiber longitudinal temperature gradient should be large during the forming of these cavities. Meanwhile, the interaction between the surrounding solid matters and the dispersive phase, probably including plasmas^4,13,37^, excited in this occasion was little evaluated experimentally. These unequilibrated environments makes theoretically modelling the forming of the cavities difficult. Nevertheless, it is indicated that *u*_0_ could influence the evolution of the fluid instability by feeding the dispersive phase, the oxygen, into the substances. Higher *u*_0_ can resist more the diffusion of the gas in the silica substances, thus compressing the oxygen together to form the long leading in-fiber cavities.

**Figure 6:**
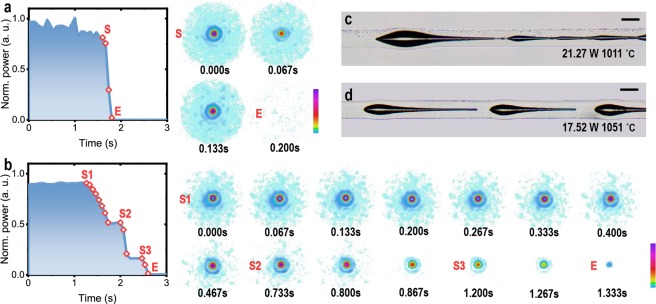
Evolution of beam profiles after the IFF. (**a**) Evolution of beam profile after IFF-with-PFF. (**b**) Evolution of beam profile after IFF-without-PFF. Shown in the left are normalized real-time powers of beam spots varying with time; 1.0 a.u. (vertical) equal the powers measured at the beginning of the tests. The red diamonds labeled on the curves correspond to the beam profiles shown right-sided in sequence. (**c**) and (**d**) Micrographs showing the cavities generated after the IFF in the cases of (**a**) and (**b**), respectively. They are made by stitching consecutively captured pictures together. At the bottom-right of the micrographs are the input powers of the fibres and the temperatures at which the IFF happened; the bars are of 130 μm length.

In literature, it was hard to observe the evolutionary process of fiber fuse. Fiber fuse emits strong and broad-spectrum irradiations, which could not be effectively filtered without hindering the observation itself (as can be seen in references^18,38^). Nevertheless, the evolutionary process of fiber fuse after the IFF here could be observed via monitoring the output beam profiles of the fibers, due to the advantage of our experimental setup. A large number of tests suggested that the evolution of beam profiles had the same pattern in all the tested fibers above. For example, Fig. 6 shows the evolving beam profiles right after the IFF in fiber 1. As can be seen, in the IFF-with-PFF, the evolution of fiber fuse in this case is too fast to be recorded by the beam profiler. The output beam spot of the fiber suddenly disappeared in Fig. 6a; the micrograph of the positions of IFF indicates a swift PFF following the IFF. In contrast, when the feeding optical power became low, some details of the evolution of fiber fuse was captured. As Fig. 6b shows, the intensity of the beam spot gradually decreased in several seconds; the micrograph indicates formation of several droplet-shaped cavities in the fibers. The evolution of fiber fuse here seemed to be discontinuous: between two plummets of the intensity was a short period of stable output that lasted for about 400 ms. This could suggest that the production of the gas was intermittent. Thus, the forming of the leading cavities could be a comprehensive effect of in-fiber fluid instability and discontinuous chemical reactions. The discontinuity can relate to the interaction between the substances and the feeding laser power under periodically oscillating temperature gradient after the IFF (which we had, however, no direct experimental method to verify). Furthermore, this phenomenon could possibly indicate a similar process observed before^18^, in which no in-fiber cavities formed in the PFF near its threshold optical power but periodical pulses of light emission happened.

The beam profiles offer a new dimension to the understanding of the evolution of fiber fuse right after the IFF. The beam profiles varied rapidly with time after the IFF. Although this phenomenon was not observed before, it could relate to the forming of the leading in-fiber cavities. A hint was that previous studies found that the light scattered by in-fiber cavities generated by PFF could change with environmental variations^39^. The time spent by the variation of the beam profiles might correspond to the geometrical distribution of the gas phase in fibers generated after the IFF. They can offer critical experimental basis, as image observations of the IFF are inapplicable, for possible theoretical models to describe the IFF. Here, it was found that the time of the optical power’s decreasing to zero after the IFF could span in a wide range from less than 1/15 seconds to more than a second in a several-millimeter-long section of fiber. It suggests that the evolution of fiber fuse is highly sensitive to the feeding optical power provided stable temperature environment for triggering the IFF. It verified that timely control of the feeding optical power may change the characteristics of the leading in-fiber cavities formed after the IFF.

## Discussion

This study originally objects determining the critical temperatures and optical powers for the IFF. The critical temperatures and critical optical powers for the IFF were determined by monitoring the temperatures and optical powers at the moment of the macroscopically observed fiber fuses that were preceded by the transient IFF. A quantitative correlation between the critical temperature and the critical optical power was revealed, which deduced an energy that very likely relates to the diffusion of oxygen in the substances of the fiber. Based on this correlation, the IFF can be mathematically predicted under given working environment. Generally, higher *u*_0_ are associated with narrower ranges of critical temperature for the IFF, as well as higher critical temperatures when the transmitted optical powers in fibers are high. The quantitative prediction of the conditions for IFF by *u*_0_ can thus direct the design of fiber systems to avoid catastrophic fiber fuse effect. This could save a large amount of economical losses as well as significantly enhance the reliability of the fiber systems in many important applications, such as high-power lasers. Moreover, as *u*_0_ possibly relates to the diffusion of oxygen, a technical route for suppressing IFF in silica fibers may emerge as increasing the energy barrier for that diffusion. So far, the complex chemical compositions of the state-of-the-art fibers still present large difficulties for verifying the influence of any single kind of dopants on the critical conditions for the IFF. Thus, validating this route requires intimate control of the chemical formulations and the production processes of fibers in future investigations. Once the critical conditions for the IFF can be raised in optical fibers, the performances of the fiber systems (such as output powers or applicable environmental temperatures) can be significantly improved to a new stage. Apart from the downside, fiber fuses also exhibit powerful ability to modify internal structures of optical fibers. Recently, using the in-fiber cavities left by the propagation of fiber fuse to make sensors has been demonstrated^40–42^. As our results suggested, the in-fiber cavities formed after the IFF could have different characteristics under different triggering conditions. In-fiber cavities can form very efficiently in IFF-without-PFF without destroying the whole optical fiber (unlike the propagation of fiber fuse otherwise). Should the critical conditions be controlled in more sophisticated ways, the IFF-without-PFF can become a new approach to fabricating in-fiber microstructures in future works.

## Methods

### Characterizing the critical temperatures and optical powers for the IFF

For each test, a fiber was processed with concentrated sulfuric acid (99.8%) and acetone to remove their polymer coating layers. Then, the fiber was pulled through the heating tube of a tube furnace. It was necessary to avoid rubbing the to-be-heated section of the fiber against the inner surface of the tube, which could inflict injuries on the fiber and thus compromise the expected conditions for IFF. Afterwards, two limestone plugs (with 3-mm-diameter perforations to let the fiber through) were installed to stabilize the temperature field in the heating tube. The length of the to-be-heated section of the fiber was 600 mm. One end of the fiber was fusion-spliced to a home-made 1064-nm continuous-wave fiber laser. A clads light stripper therebetween was made by immersing the fusion-splicing-spot and its 80-mm-long vicinity into high-refractive-index greases. It would minimize the influence of possible cladding light on the IFF, which were introduced by different numerical apertures among the fibers. During each test, a new optical power was input into the tested fiber and maintained. The heating process began at room temperature. Maximum heating rate (around 10 °C/min at over 800 °C) of the tube furnace was used for every test. This was to minimize the influence of deterioration, including collapse of refractive-index profiles, of the fiber in high temperatures. Calculations show that there could be no significant dopant diffusion during the heating process before the IFF; the temperatures used was expected to result in maximum dopant diffusion rates of less than 10^−17^ m^2^/s^43^. Thus, the heating setup could impose no significant influence on the final results. Besides the radial temperature gradients detailly verified around Figs 2 and 3, there was also an expected temperature gradient that was very trivial and axially symmetrical in along the heating tube. This trivial temperature gradient could be neglected in the estimation of the assumed distributed absorption later in the determination of the critical optical powers. However, it could still be captured by Eq. 1 that sensitively depends on the critical temperatures, as the IFF always happened around the midpoints of the heated section of fibers. This experimental result also validated the heating setup: since the IFF always capture the point that first reaches the critical temperatures, how the temperature gradients are in the rest part of the heated fiber will be not relevant. That is, using other heated lengths of fiber hypothetically will still obtain the same experimental conclusions as here.

### Numerical simulation to validate the temperature

Since it was hard to evaluate the exact amount of absorption in the experiment, we let the 1-W-power be the heat source of the core of the heated fiber. This value was much larger than that in the real situation; it should help evaluate the maximum difference between the real temperatures of fiber and the measured values in experiment. Considering the uniform temperature field inside the furnace, the absorption could be presumed uniform in the heated fiber. Therefore, a heat source was applied in the core of the fiber being 2.12 × 10^10^ W/m^3^, which corresponded to an assumed absorption of 1 W in the core of the 600-mm heated fiber. The radius of the heating tube was 30 mm in the experiment. This model was solved in 3D space using a commercial software (COMSOL Multiphysics). A solid heat transfer model was used, which neglected the effect of thermal convection in air. Nevertheless, this treatment should give accurate enough results in the small space inside the tube furnace. The solution used the built-in materials: the average silicate and the air.

### Determination of the critical optical power ***P***_***c***_

In the experiment, the measurable optical powers were the input powers *P*_*in*_ and the output powers *P*_*out*_ of the fibers. Considering that the leakage (including the trivial distributed absorption) verified in the above text around Fig. 3 was nearly uniformly distributed along the heated fiber, the relation between *P*_*out*_ and *P*_*in*_ can be described by *P*_*out*_ = *P*_*in*_exp(−*αL*), where *L* is the length of the heated fiber and *α* represents the power loss. The critical optical powers for the IFF should be the optical powers that passed through the positions of the IFF. That is, *P*_*c*_ = *P*_*in*_exp(−*αL*/2). Therefore, we can transform the equation into ${P}_{c}=\sqrt{{P}_{in}{P}_{out}}$. Here, we used the averages of the output powers recorded in the last 3 °C before the IFF as *P*_*out*_ in Fig. 4 and Supplementary Tables S1–S4 for reducing the influence of the fluctuations of the output powers on the values of *P*_*out*_. Additionally, as the differences between the experimental values of *P*_*in*_ and those of *P*_*out*_ were very small, this determination of *P*_*c*_ was, in fact, of high robustness. That is, replacing *P*_*c*_ with *P*_*in*_, *P*_*out*_ or (*P*_*in*_ + *P*_*out*_)/2 could neither influence the conclusions. This also suggests that the heating method of this study, heating a section of fiber instead of a point on fiber, was applicable.

### Observing the in-fiber cavities and the evolution of beam profiles

The positions of the IFF could be easily located by naked eyes or finger-smoothing the fibers in most cases, as large in-fiber cavities would form after the IFF at the beginning of the paths of fiber fuse. Thereby, their relative positions in the heating tube could be inferred. When taking the micrographs, the fibers were immersed in refractive-index matching fluid of approximately 1.47 refractive-index to mitigate distortion and enhance contrast of the images. For observing the evolutionary process of the beam profiles, the power meter in Fig. 1a was replaced by a beam profiler. The software (BeamStar) working with the beam profiler did not support recording of real-time data; thus, the profiles were recorded by screenshots. The used time resolution of the profiler was the highest (15 fps) and the same as that of the screenshots. The IFF could be predicted by using the mathematical correlation revealed in Fig. 4e. Therefore, it was only needed to record the beam profiles in the first (to determine the initial intensity of beam spots in Fig. 6) and the last several minutes in each test.

## Supplementary information

Exploring the initiation of fiber fuse

## Data Availability

All the data used to support the findings of this paper are available from the corresponding author upon requests.
