# Supplementary material for: Exploring the initiation of fiber fuse
Source: Sci Rep. 2019 Aug 12;9:11655. doi: 10.1038/s41598-019-47911-0 (PMC6690914; doi:10.1038/s41598-019-47911-0)
Supplement: Supplementary file 1 — Exploring the initiation of fiber fuse [file 41598_2019_47911_MOESM1_ESM.docx]

**Supplementary Information**

**Exploring the initiation of fiber fuse**

Qirong Xiao, Jiading Tian, Ping Yan*, Dan Li & Mali Gong

**Supplementary Notes**

**1. Specifications and characteristics of the tested fibers.** All the tested fibers in this study are commercially available products. The four kinds of active (Ytterbium-doped) fibers are numbered 1-4 respectively; their product specifications, provided by the suppliers, are shown in Supplementary Table S5. The IFF were successfully triggered in these fibers. The two kinds of passive (Germanium-doped) fibers are numbered 5 and 6. Fiber 5 is a GDF-10/130 fiber of core diameter 10.9 μm and core NA=0.08; fiber 6 is a standard G652.D fiber of core diameter 8.2 μm and core NA=0.14. But no IFF happened to these fibers in the experiment. The chemical compositions of the tested fibers can be also implied by the results in Supplementary Note 6 and Supplementary Table S6.

In the experiment, we use optical power as one of the critical conditions for the IFF. Practically, it has no difference with using optical power density, which was a parameter of experiment in some previous studies. The used optical power densities in our fibers 1-6 could be roughly estimated using their core diameters. However, previous studies were used to compare optical densities calculated by mode field diameters. The mode field diameters at our experimental laser wavelength 1064 nm were not provided by the suppliers. Nonetheless, they could be calculated using the core diameters and numerical apertures of the fibers. Two typical equations, $V=\left( {2\pi}/{\lambda_{0}} \right)r_{c}NA$ and $s_{0}=r_{c}\left( 0.65+1.619V^{-2/3}+2.879V^{-6} \right)$, ^[1]^ were used to calculate the mode field radius $s_{0}$ (half of mode field diameter). The calculated mode field radii for fibers 1-6 were respectively 6.16, 5.74, 6.63, 6.63, 5.72 and 3.73 μm around the laser wavelength 1064 nm. The optical power densities were calculated using the standard of previous studies, which was dividing the optical powers with the mode field area $\pi s_{0}^{2}$. The corresponding maximum optical power densities used were 50.6, 58.9, 43.8, 43.7, 58.4 and 114.4 MW/cm^2^ respectively for fibers 1-6. (due to significant mismatch between fiber 6 and the output fiber of our home-made fiber laser, the maximum optical power that entered fiber 6 was only 50.1 W, while that entered the other fibers all exceeded 60 W, as shown in Supplementary Tables S1-S4).

**2. Electron spin resonance (ESR) test and inductively coupled plasma (ICP) mass spectrometry (MS) test of the fibers.** In literature, ESR was used to study defects in amorphous silica. Some previous studies reported that ESR signals suggested Ge-related defects inside Ge-doped single-mode optical fibers ^[2]^. Despite that how the ESR resonances was specifically attributed to Ge was not mentioned at all, this result is intuitively right, as the IFF must have created many broken bonds in the substances of the fibers.

Considering that the fibers that the IFF was triggered in are all Ytterbium-doped fibers, we performed ESR tests on one of them (the fiber 3) to verify the formation of defects. We tested both pre-fiber-fuse (pristine) and post-fiber-fuse (PFF-damaged) samples of fiber 3. Each sample was made by cutting 3-metre-long fiber into pieces with each piece being around 3 centimeters. The pieces were put into the ESR instrument (JEOL FA-200). A standard manganese marker was used to calibrate g values; blank sample signal was compensated before testing the fiber pieces. The used microwave frequency was 9060 MHz, power 0.1 mW, and magnetic field from 315 to 330 mT. Under these parameters the signals were not saturated. Liquid nitrogen was used to perform the ESR at 0 ˚C and -168 ˚C.

The results are shown in Supplementary Fig. S1. As can be seen, after the PFF, strong paramagnetic resonances arose between g values 2.0000 and 2.0030. The main resonance peaks found in the PFF-damaged fibers seem to have already existed in the pristine fibers. It suggests that the types of defects responsible to these resonances may belong to the intrinsic defects in the substances. However, it is hard to tell the kinds of these defects based on the spectra alone. The spectra indicate that there were plenty of kinds of defects in the fibers. This may relate to the complex chemical formulations of the fibers.


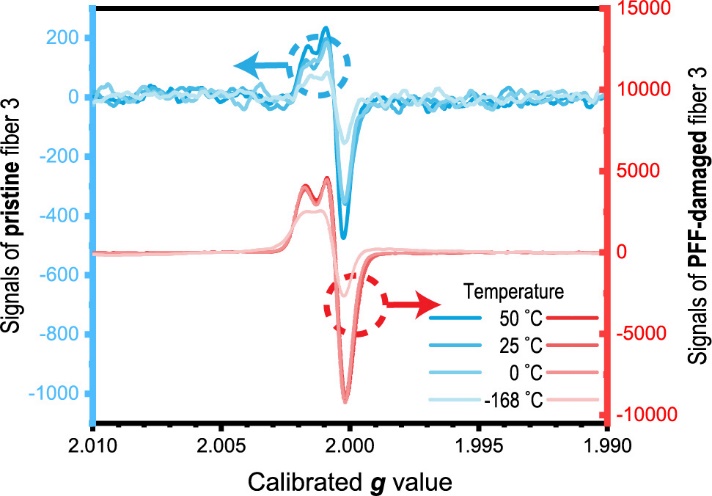


**Fig. S1**. **ESR spectra of the pristine and the PFF-damaged fiber 3.**

We performed ICP MS tests (Thermo Fisher iCAP Q) to all the tested fibers. The sample of each fiber was weighted (around 20 mg) and placed in a polytetrafluoroethylene (PTFE) beaker. The digestion of the sample was finished with MOS-class hydrofluoric acid and nitric acid in 180 ˚C. The dilution of the sample used high purity water by Millipore. The preliminary results found multiple elements in the fibers. The roles of many of the elements and their interactions in the environment of the fibers are yet to be verified. The spatial distributions of these elements in the cross section of the fibers are also unclear. We quantitatively calculated the mass fraction of ten elements, the results are shown in Supplementary Table S6. We have not found a simple dependence of the parameter $u_{0}$ on the concentration of any single element. As can be understood, verifying the physical link relies on intimate knowledge of the production processes of respective fibers. But it can be ascertained that little Germanium existed in the tested active fibers.

**3. Differences and connections with previous studies on the explanation of IFF.** Previous studies ^[2-4]^ considered that defects in the substances of fibers could relate to the optical absorption that was assumed responsible for the inevitable realization of fiber fuse, which had a similar position with our definition of IFF. The studies did not elaborate on rigorous proofs indicating any specific kinds of physical processes be responsible for the IFF. The defects were vaguely described as germanium defects, whose concentration was presumed to be proportional to the optical absorption. Thus, the absorption $\alpha$ could be written as $\alpha=\alpha_{0}\exp\left( -{E_{a}}/{k_{B}T} \right)$, where $E_{a}$ was the presumed activation energy and $\alpha_{0}$ a coefficient in inverse unit of length. The form of this equation, being like that of Eq. 2 in this study, could have made one think that they were the same and that so did the theory in those studies and the correlation revealed in this study here.

However, we should notice that they are very different. In the previous studies, the theory was established on complex calculations of the speed of PFF to approximate the experimentally measured data. Therein, the relation $\alpha=\alpha_{0}\exp\left( -{E_{a}}/{k_{B}T} \right)$ was used as an interchangeable conjecture (a step-profile absorption $\alpha=\alpha_{0}\left( T-T_{0} \right)$ can also produce similar results ^[5]^) to represent the heat source in the model of calculation. The parameters $\alpha_{0}$ and $E_{a}$ were artificially assigned as 2.2 eV ^[2,4]^ or 2.5 eV ^[3]^ for unknown $E_{a}$; $\alpha_{0}$ was $1.2\times{10}^{7}$/m ^[2]^, $1.06\times{10}^{8}$/m ^[3]^ and $4.8\times{10}^{6}$/m ^[4]^ for the absorption caused by the defects. A slightly different set of other parameters will eventually require 13.6 eV ^[6]^ for a similar concept of energy in the algorithms. As can be seen, in this conjecture, the values of $E_{a}$ can be highly uncertain without experimental bases. Besides, none of the previous studies paid interests for the critical conditions that trigger the IFF. The correlation between the critical conditions was neither experimentally revealed nor theoretically predicted as $E_{a}$ was introduced.

It is noteworthy that we have no intention to question the details of the calculations. In fact, the assumptions in the previous model are so far powerful tools to explain fiber fuse and acquire some degree of foresight of the phenomenon. The point we are trying to make is that the previous studies are intrinsically different from this study in both methodology and conclusions, despite some of them involved $E_{a}$, which looked like the parameter $u_{0}$ in this study. In this study, $u_{0}$ is determined only by experimental results. Meanwhile, the ICP-MS results in Supplementary Table S6 reveal the overwhelming difficulty in theoretically modelling (or even assuming) the exact chemical changes in the substances of the fibers nowadays. This complex in chemical formulations is so far less considered in the use of the optical fibers. It is found that in the tested fibers in which IFF happened, no germanium was detected; conversely, the fibers containing germanium survived in 1200 ˚C without IFF. These facts all refute a direct physical link between $u_{0}$ and germanium themselves. Considering the experimental values of $u_{0}$, the IFF should be more pertinent to a diffusion of oxygen in the silica reduction process. Although this link must require further experimental verifications, it is critical for establishing a right theoretical model for the IFF. Nevertheless, at this point, we either lack the access to information on the chemical formulations and production processes of the fibers, as the previous studies ^[4]^ admitted. Therefore, achieving the goal will require more researchers who have that access engage.

**Supplementary Tables**

**Table S1.** Data of the IFF in fiber 1.

| $P_{in}$ / W | $P_{out}$ / W | *T* / ˚C | $P_{in}$ / W | $P_{out}$ / W | *T* / ˚C |
| --- | --- | --- | --- | --- | --- |
| 61.0 | 59.5 | 836 | 24.78 | 23.28 | 978 |
| 56.7 | 55.2 | 846 | 21.83 | 20.17 | 996 |
| 53.2 | 51.7 | 857 | 19.67 | 17.86 | 1024 |
| 47.72 | 46.28 | 867 | 17.81 | 15.76 | 1045 |
| 43.96 | 42.46 | 873 | 17.61 | 15.40 | 1051 |
| 38.75 | 37.27 | 892 | 17.27 | 14.76 | 1056 |
| 34.85 | 33.58 | 910 | 16.77 | 14.28 | 1063 |
| 29.70 | 28.19 | 940 | 16.33 | 13.56 | 1072 |

**Table S2.** Data of the IFF in fiber 2.

| $P_{in}$ / W | $P_{out}$ / W | *T* / ˚C | $P_{in}$ / W | $P_{out}$ / W | *T* / ˚C |
| --- | --- | --- | --- | --- | --- |
| 61.3 | 60.7 | 869 | 28.65 | 27.22 | 952 |
| 56.6 | 55.8 | 875 | 24.52 | 23.10 | 965 |
| 52.7 | 51.9 | 883 | 21.46 | 19.53 | 973 |
| 47.11 | 46.24 | 892 | 20.14 | 18.19 | 984 |
| 42.58 | 41.07 | 901 | 19.49 | 17.23 | 990 |
| 37.68 | 36.47 | 920 | 19.09 | 16.39 | 997 |
| 32.56 | 31.33 | 938 | 18.71 | 15.82 | 1001 |

**Table S3.** Data of the IFF in fiber 3.

| $P_{in}$ / W | $P_{out}$ / W | *T* / ˚C | $P_{in}$ / W | $P_{out}$ / W | *T* / ˚C |
| --- | --- | --- | --- | --- | --- |
| 60.9 | 59.8 | 958 | 28.43 | 26.65 | 1008 |
| 56.4 | 55.2 | 960 | 24.20 | 22.15 | 1026 |
| 52.4 | 51.1 | 967 | 20.92 | 18.95 | 1036 |
| 47.28 | 46.04 | 974 | 19.96 | 17.61 | 1040 |
| 41.21 | 40.09 | 981 | 19.58 | 16.90 | 1043 |
| 37.41 | 36.22 | 988 | 19.21 | 16.11 | 1050 |
| 33.61 | 32.22 | 995 | 18.76 | 15.44 | 1054 |

**Table S4.** Data of the IFF in fiber 4.

| $P_{in}$ / W | $P_{out}$ / W | *T* / ˚C | $P_{in}$ / W | $P_{out}$ / W | *T* / ˚C |
| --- | --- | --- | --- | --- | --- |
| 60.9 | 59.9 | 973 | 28.76 | 27.56 | 1030 |
| 57.0 | 56.0 | 976 | 24.24 | 22.95 | 1041 |
| 52.3 | 51.4 | 980 | 20.27 | 18.80 | 1064 |
| 47.03 | 46.43 | 985 | 18.69 | 16.94 | 1072 |
| 42.16 | 41.38 | 995 | 18.22 | 16.14 | 1078 |
| 37.71 | 36.75 | 1003 | 17.70 | 15.41 | 1085 |
| 32.67 | 31.38 | 1016 |  |  |  |

**Table S5.** Specifications of the tested active fibers 1-4.

| Fiber Number | 1 | 2 | 3 | 4* |
| --- | --- | --- | --- | --- |
| Working wavelength / nm | 1060 | 1050 | 1060 | 1060 |
|  | 1115 | 1090 | 1115 | 1115 |
| Core diameter / μm | 11.9 | 11.0 | 10.9 | 11.6 |
| Inner-clad diameter / μm | 131.1 | 134.2 | 129.2 | 126.9 |
| Coating diameter / μm | 246.9 | 246.2 | 249.3 | 243.3 |
| Concentricity / μm | 0.31 | 0.7 | 1.1 | 1.1 |
| Inner-clad NA | 0.46 | 0.46 | 0.46 | 0.46 |
| Core NA | 0.075 | 0.08 | 0.065 | 0.065 |
| Clads absorption at 915 nm / dB/m | 1.31 | 1.35 | 1.04 | N.A.* |
| Clads absorption near 976 nm / dB/m | 3.9 | 3.53 | 3.01 | N.A.* |

*N.A., not available from the suppliers.

**Table S6.** Mass fraction of some dopants in the tested fibers measured by ICP MS.

| Unit | Fiber Number | | | | | |
| --- | --- | --- | --- | --- | --- | --- |
|  | _ Active fibers _ | | | | Passive fibers | |
| mg/kg | 1 | 2 | 3 | 4 | 5 | 6 |
| 7.Li | 2.86 | 4.77 | 2.07 | 1.44 | 1.14 | 1.92 |
| 11.B | 5.97 | N.D.* | 6.91 | 5.48 | N.D.* | 1.79 |
| 24.Mg | 201.53 | 238.78 | 7467.50 | 19045.14 | 293.58 | 199.09 |
| 27.Al | 227.84 | 142.12 | 367.38 | 354.04 | 175.09 | 112.43 |
| 48.Ti | 131.35 | 422.31 | 118.92 | 119.03 | 166.98 | 184.19 |
| 73.Ge | N.D.* | N.D.* | N.D.* | N.D.* | 153.58 | 295.86 |
| 88.Sr | 16.75 | 41.43 | 22.73 | 4.05 | 98.02 | 21.75 |
| 89.Y | 6.18 | 12.78 | 54.59 | 129.31 | 11.53 | 8.24 |
| 90.Zr | 2.53 | 0.65 | 275.24 | 758.2 | 5.52 | 2.29 |
| 172.Yb | 70.91 | 46.8 | 94.37 | 97.53 | 0.47 | 0.13 |

*N.D., not detected. The concentrations of the elements are below the limit that they can be distinguished from the experimental environment in the instrument.

**Supplementary references**

1. Snyder, A. W.; Love, J. D. *Optical waveguide theory* (Chapman and Hall, 1983).
2. Hand, D. P. & Russell, P. S. Solitary thermal-shock waves and optical-damage in optical fibers - the fiber fuse. *Opt. Lett.* **13**, 767-769 (1988).
3. Shuto, Y.; Yanagi, S.; Asakawa, S.; Kobayashi, M. & Nagase, R. Evaluation of high-temperature absorption coefficients of optical fibers. *IEEE Photonics Technol. Lett.* **16**, 1008-1010 (2004).
4. Facao, M.; Rocha, A. M. & Andre, P. S. D. Traveling Solutions of the Fuse Effect in Optical Fibers. *J. Lightwave Technol.* **29**, 109-114 (2011).
5. Ankiewicz, A.; Chen, W.; Russell, P. St. J.; Taki, M. & Akhmediev, N. Velocity of heat dissipative solitons in optical fibers. *Opt. Lett.* **33**, 2176-2178 (2008).
6. Shuto, Y. Cavity Formation Modeling of Fiber Fuse in Single-Mode Optical Fibers. *Adv. Optoelectron.* **2017**, 5728186 (2017).
